# Direct-to-Implant Breast Reconstruction As a Single-Stage Operation in the Pre-Pectoral Plane: Review of an Institutional Experience

**DOI:** 10.1093/asjof/ojaf018.002

**Published:** 2025-05-13

**Authors:** Chris Amro, Carter Boyd, Kshipra Hemal, Thomas Sorenson, Jenn Park, Nicholas Vernice, Oriana Cohen, Mihye Choi, Nolan Karp

**Affiliations:** NYU Langone Health, New York, NY; NYU Langone Health, New York, NY; NYU Langone Health, New York, NY; NYU Langone Health, New York, NY; NYU Langone Health, New York, NY; NYU Langone Health, New York, NY; NYU Langone Health, New York, NY; NYU Langone Health, New York, NY; NYU Langone Health, New York, NY

## Abstract

**Goals/Purpose:**

With improvement in mastectomy techniques, rates of single-stage direct-to-implant (DTI) breast reconstruction have risen throughout the United States. DTI reconstructions avoid placement of a temporizing tissue expander and the subsequent expansion process which requires frequent visits prior to final insertion of the permanent prosthesis. Several studies in the literature have called to question if DTI reconstruction can be appropriately called a “one and done” operation citing high re-operation rates. The objective of this study was to classify reoperation rates for DTI and two-stage prepectoral implant-based breast reconstruction (IBBR).

**Methods/Technique:**

A retrospective review was performed of all consecutive, prepectoral breast reconstructions between 2017-2024 at a single institution. Variables of interest included patient demographics, operative characteristics, complication rates, and reoperation rates. Major complications included any complication involving the breast that required readmission or reoperation while minor complications included any breast complication requiring outpatient antibiotics, procedure, or wound care. Reoperations for aesthetic improvement were classified separately.

**Results/Complications:**

A total of 552 breasts (333 patients) were included. The study population represented 323 two-stage IBBRs and 229 DTI reconstructions. Mean patient age was 51 years, with a significantly higher BMI in the two-stage cohort compared to the DTI cohort (27.5 vs 25.7 kg/m²). A greater proportion of DTI reconstructions were performed in patients with prophylactic mastectomies (59.4% vs. 33.7%, p<0.05). Mean mastectomy weight was 616.1 ± 460.3 grams and was significantly lower in the DTI cohort (561.1g ± 445.6 vs 653.4g ± 467.1, p<0.05). Major complications occurred in 80 breasts (14.5%), with a significant difference observed between cohorts (19.8% in the two-stage cohort vs. 7.0% in the DTI cohort, *p* < 0.05). Minor complications occurred in 147 breasts (26.6%) and were more frequent in the two-stage cohort (30.7%) than in the DTI cohort (21.0%, *p* <0.05). The overall reoperation rate for aesthetic improvement was higher for DTI compared to two-stage cohort (15.3% vs 5.3%, p<0.05). Patients in the DTI cohort also had a significantly higher rate of revisional fat grafting (11.4% vs. 3.4%, p<0.05). There were no significant differences regarding revisions for mastopexy, dogears, or scars (p>0.05).

**Conclusion:**

This study highlights that prepectoral DTI reconstruction has low reoperation rates, with significantly fewer major and minor complications compared to two-stage IBBR. While reoperations, if needed, were more often for aesthetic reasons in the DTI group, the overall number of procedures was still lower than in the two-stage cohort. Patients can be counseled that DTI reconstruction offers a safe and aesthetically favorable option, with a reduced total number of surgeries and lower complication rates compared to traditional two-stage reconstruction.

